# Modification of Cardiovascular Drugs in Advanced Heart Failure: A Narrative Review

**DOI:** 10.3389/fcvm.2022.883669

**Published:** 2022-05-23

**Authors:** Manuel Martínez-Sellés, Tomasz Grodzicki

**Affiliations:** ^1^Servicio de Cardiología, Hospital General Universitario Gregorio Marañón, CIBERCV, Universidad Europea, Universidad Complutense, Madrid, Spain; ^2^Department of Internal Medicine and Gerontology, Jagiellonian University Medical College, Krakow, Poland

**Keywords:** advanced heart failure, palliative care, cardiovascular drugs, drug withdrawal, prognosis, end of life

## Abstract

Advanced heart failure (HF) is a complex entity with a clinical course difficult to predict. However, most patients have a poor prognosis. This document addresses the modification of cardiovascular drugs in patients with advanced HF that are not candidates to heart transplantation or ventricular assist device and are in need of palliative care. The adjustment of cardiovascular drugs is frequently needed in these patients. The shift in emphasis from life-prolonging to symptomatic treatments should be a progressive one. We establish a series of recommendations with the aim of adjusting drugs in these patients, in order to adapt treatment to the needs and wishes of each patient. This is frequently a difficult process for patients and professionals, as drug discontinuing needs to balance treatment benefit with the psychological adaption to having a terminal illness. We encourage the use of validated assessment tools to assess prognosis and to use this information to take clinical decisions regarding drug withdrawal and therapeutic changes. The golden rule is to stop drugs that are harmful or non-essential and to continue the ones that provide symptomatic improvement.

## Introduction

According to the World Health Organization Global Atlas of Palliative Care at the End of Life there are already more adults in need of palliative care due to cardiovascular conditions than to cancer^1^ and population aging will probably increase this tendency (1), making essential to include palliative approach in many routine cardiology consultations. Disabling symptoms are common in patients with cardiovascular disease, particularly in those with advanced heart failure (HF). The current Heart Failure Association-European Society of Cardiology criteria for defining advanced HF include severe and persistent symptoms (functional class III or IV), severe cardiac dysfunction, recent (in the last 12 months) episodes of congestion requiring high-dose intravenous diuretics, low output requiring inotropes or vasoactive drugs or malignant arrhythmias, and severe impairment of exercise capacity (2).

Deprescribing is a growing problem in modern medicine, which has been dominated by treatments focused on treating specific disease entities rather than treating patients with a global approach. Evidence-based medicine is mainly based on clinical trials results which included carefully selected patients with a specific disease entity. Patients with comorbidities, advanced age or disability are frequently excluded from the randomized trials. This is very relevant in HF, as mean age in HF patients is about 80 years and comorbidities are the rule. Guidelines for dealing with specific conditions as HF, mostly built on the results of clinical trials, rarely focus on the problem of multimorbidity and polypharmacy and the need to assess drugs interactions used for multiple indications. Previous authors have suggested that this is the case in HF, as current guidelines for diagnosis and treatment do not fit with clinical complexity (3). HF is rarely an isolated condition and in most cases occurs together with diseases of the cardiovascular system, such as arterial hypertension, ischemic heart disease, valvular disease, or atrial fibrillation; metabolic diseases such as diabetes or gout; respiratory disorders such as chronic obstructive pulmonary disease; chronic kidney disease; or liver failure. As a result, a patient with advanced HF is frequently treated by several specialists, each of whom treats “their” disease, leading to polypharmacy and common side effects and drug interactions (4).

In this context, reconsidering drugs use in the last phase of life seems obvious but, as this strategy is inadequately described in guidelines, protocol procedures for deprescribing are rarely launched. Patients with advanced HF often take multiple medications that may not have beneficial effects in view of their limited life expectancy and changing organ function. The potential interactions between cardiovascular drugs and palliative symptoms relievers should also be considered. On the other hand, stopping all cardiovascular medications is frequently a mistake as some improve symptoms and should be maintained while tolerated. In any case, decisions about the discontinuation of preventive medicines for individuals approaching the end of life are increasingly complicated, by the lack of clear deprescribing guidelines for these medicines (5). However, most data suggest that deprescribing should be more frequent, for instance, the five most common classes of medications prescribed near the end of life are antihypertensives, broncholytic drugs/bronchodilators, laxatives, antidepressants, and gastric protection agents (6).

Limiting polypharmacy decreases the risk of adverse effects, medical errors, associated cost and harmful drug interactions. Moreover, the time lag to benefit from the use of many cardiovascular drugs is frequently longer than the life expectancy of patients with advanced HF that are not candidates to heart transplantation or ventricular assist device. In these patients, frequently there is a need to modify, and even to discontinue, cardiovascular drugs. The decision to discontinue some drugs like lipid-lowering products is rather straightforward. In other cases, like antithrombotic therapy and specific HF drugs, the medication should be stopped in some patients but not in others. For instance, discontinuation of some HF drugs may provoke exacerbation of symptoms and should be considered only in the last weeks of life. In this revision, we address the modification of cardiovascular drugs in patients with advanced HF who are not candidates to life-prolonging therapies. We encourage the use of validated assessment tools to assess prognosis and to use this information to take clinical decisions regarding drug withdrawal and therapeutic changes. Physiotherapy and rehabilitation should also be recommended for all HF patients, as they may prevent or diminish pain and fatigue. In addition, the psychological effects of exercise cannot be forgotten.

## Palliative Effects of Cardiovascular Drugs

Dyspnea is a common complaint and even during hospital admission, breathlessness is frequently under-diagnosed and under-treated (7). Moreover, about a quarter of patients admitted with HF persist with severe dyspnea at discharge and HF patients present worse quality of life than patients with respiratory diseases (8). Diuretics are the basis for treatment of dyspnea and are used for pulmonary decongestion. The route of administration and dosages should be consistent with the clinical situation and degree of congestion, with close monitoring of the patient's response. In ambulatory patients with advanced HF, an alternative may be to administer subcutaneous furosemide (9). Oxygen therapy is also important to relive dyspnea, particularly in hypoxemic patients. Vasodilators can also be used as support in the treatment of dyspnea but can cause symptomatic hypotension or worsening of the renal function. The use of drugs to improve breathlessness should be complemented with other simple and effective measures, such as fresh air (breezes or fans) directed toward the patient's face. In addition, in case of refractory breathlessness, pharmacologic interventions are mandatory, and opioids play a key role in this setting. We need to remember that morphine has cardiovascular effects due mainly to its vasodilatory properties and effects related to anticipated anxiolysis. However, although morphine does not seem to increase the risk of short-term death in patients with acute advanced HF, the risk of long-term death and invasive ventilation might be increased (10).

Leg pain and discomfort related to edema can be frequently observed in patients with right ventricular HF or tricuspid valve insufficiency and might be exacerbated by HF-related liver cirrhosis and hypoalbuminemia. Loop diuretics and aldosterone antagonists are useful, although an equilibrium between their benefits and the risk related to hypovolemia or, in the case of loop-diuretics, hypokaliemia should be scrutinized. Also, diuretic resistance despite escalating doses of a loop diuretic to a ceiling level (80 mg of furosemide once or twice daily or greater in those with reduced glomerular filtration rate) is common in these patients. The use of local leg compression can also be considered (11). On the other hand, aldosterone antagonists may lead to hyperkaliemia or renal failure.

Fatigue is also common in patients with advanced HF and might be their main symptom. Ambulatory inotropes are an option in some patients that is increasingly offered as a palliative therapy. Although there remains a profound lack of data and guidance on the effect of palliative inotropes on quality of life, limited available data suggest that inotrope therapy improves functional class and does not impact survival (12). Levosimendan has shown some advantages: for instance, its effects are sustained after the initial infusion, it can be used in patients treated with beta-blockers, and it does not increase oxygen requirements (13).

## Prognosis Assessment

The use of HF risk scores is recommended to avoid the frequent overoptimistic subjective appreciation of prognosis (14). Physicians are inaccurate in their expected life predictions for terminally ill patients and the error is systematically optimistic, thus affecting the quality of care given to patients near the end of life. None of the contemporary risk scores shows a clear superiority over the others (15). Barcelona Bio-Heart Failure (BCN-Bio-HF) calculator provides the best discrimination and overall performance but tends to overestimate the risk. Meta-Analysis Global Group in Chronic Heart Failure (MAGGIC-HF) has the best calibration, and Seattle Heart Failure Model (SHFM) and PARADIGM Risk of Events and Death in the Contemporary Treatment of Heart Failure (PREDICT-HF) tend to underestimate the risk. All these prognostic risk calculators are available and have been critically reviewed, although prognostication on an individual basis remains challenging. An uncertainty in prognosis prediction is almost inherent to advanced HF and should be explained to the patient and the family. Such previous explanation helps patients and relatives to better understand therapy re-adjustment that, as we will see in the next paragraph, might include restarting withdrawn drugs.

## Medication Review and Drug-Disease Interactions

A full medication review of all patients with advanced HF should be undertaken with a view to rationalizing medications with questionable benefit in the face of limited prognosis. Target groups of medication to consider deprescribing would usually include statins, but also drugs with long-term effects prescribed for comorbid conditions such as vitamins, bisphosphonates and proton pump inhibitors. Drug-disease interactions are common for patients with advanced HF due to changes in pharmacokinetics as a result of impaired renal function, reduced hepatic metabolism, and gut edema.

## Cardiovascular Drugs Discontinuation and Re-adjustment of Therapy

Reconsidering drugs in the last phase of life should be done more frequently, even more the case of advanced HF, as these patients frequently improve and deteriorate, sometimes unexpectedly. If patients improve, they may need new cardiovascular drugs and, in these cases, the withdrawing will be of palliative care drugs (for instance, opioids after dyspnea improvement). This re-adjustment of therapy might also include restarting or increasing previously reduced doses of cardiovascular drugs (16).

Drug deprescribing should be a proactive, patient-centered approach that should include a frequent assessment of goals of care, values, preferences and life expectancy. Patients may be reticent to the withdrawal of previously indicated therapies. Therefore, professionals need to motivate change by emphasizing the benefit of withdrawing some drugs, like the reduction in the number of pills per day or the prevention of side effects. In addition, deprescribing should not be associated to a reduction in medical care and should focus on realistic treatment goals mainly aimed quality of life improvement.

**Figure 1:**
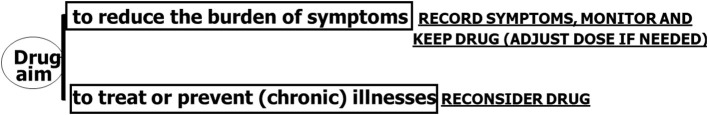
Decision tree algorithm regarding the adjustment of cardiovascular drugs in patients with advanced heart failure.

Transition of the goals of care toward improving comfort and focusing on alleviating symptoms requires a continuous communication with patients and their families. The validity of former indications for their use, after setting new goals, should be evaluated. Treatments relevant for symptom management (or prevention) should be continued unless relevant side effects appear. On the other hand, cardiovascular drugs prescribed for conditions that are becoming no longer relevant should be considered for withdrawal. Therapies causing adverse effects and most preventive drugs, especially those with a long delay in showing their benefits should be stopped. The simple message would be to maintain drugs that reduce the burden of symptoms (while recording these symptoms) and to reconsider drugs used to treat or prevent (chronic) illnesses (Figure 1). Therefore, routinely stopping cardiovascular drugs when starting palliative care is inappropriate, as many drugs are important for symptoms control.

## Anticipatory Prescribing and Deprescribing

Maintaining patient and family autonomy, preparing them for sudden unpredictable situations and providing the means for self-care are essential elements for successful care of patients with advanced HF. Anticipatory prescribing tries to prepare for future situations by doing prescriptions of drugs that might be needed in emergencies in the future. Making those drugs accessible at home with clear instructions for their use, can empower patients and caregivers in self-management until professional care is available. In a similar way, clear indications as when to withdraw a drug might help patient that are at home to stop using drugs that are no longer useful or that have become unnecessary.

## Triggers for Drug Adjustment and Bad News Delivery

Drug adjustment and deprescribing should be a gradual process initiated as HF progresses. Prognostic tools, symptom assessment tools, and events as hospitalization might be an excuse to start conversations about the goals of care, and the need to readjust drug therapy. Other triggers to initiate this conversation might be uncontrolled symptoms, recurrent HF exacerbation, progressive frailty, patient and/or caregiver concerns. The moment when a do-not-resuscitate order protocol is decided might also be a good opportunity to initiate this talk (17). However, do-not-resuscitate orders, also known as do-not-attempt-resuscitation are frequently implemented in a very advanced situation, so drug adjustment should be ideally done weeks or months before (18, 19).

Table 1 describes the six-step protocol for delivering bad news SPIKES. Although originally described for cancer patients (20) it is perfectly applicable to initiate a conversation regarding deprescribing and other palliative care issues in advanced HF (21). The setting is very important and patient privacy should be respected but, at the same time, it is essential to involve significant others unless the patient is opposed to their presence. Asking patients about their expectations and preferences regarding information before delivering it is also needed. A realistic approach of those expectations will help patients to accept drug adjustment, as will the use of clear language and the progressive presentation of the expected prognosis. Empathy helps to develop a meaningful relationship with the patient and summarizing the information with the specific therapeutic changes that will be performed gives patients and families the opportunity to voice concerns and ask questions.

**Table 1:** A six-step protocol for delivering bad news.

**Setting**
• Privacy
• Involve significant others
• Sit down
• Look attentive and calm
• Adopt listening mode
**Perception**
• Before you tell, ask
• Assess the gap between the patient's expectations and the actual medical
situation
**Invitation**
• Do not assume that all patients want to know all
• Ask about preferences regarding information
**Knowledge**
• Give a warning that bad news is coming
• Give the information in small chunks
• Use clear language
**Empathy**
• Acknowledge and address the patient's emotions
• Let them know that showing emotion is normal
**Strategy and summary**
• Ensure that the patient understands the information
• Summarize the information and give an opportunity for the patient to voice
concerns

*Please note the mnemonic acronym SPIKES [Sobanski et al., (16)]*.

## Common Cardiovascular Drugs Adjustments

Table 2 shows the most common adjustment of cardiovascular drugs in patients with advanced HF. Drugs that produce symptoms relief, as diuretics, should be kept unless relevant side effects appear. Drugs with long-term effects should be withdrawn. In some cases, the decision is not straightforward, and specific patient characteristics like renal function and blood pressure might influence the decision to continue or withdraw, for instance with angiotensin-converting enzyme inhibitors, angiotensin receptor blockers, mineralocorticoid receptor antagonists, and sodium–glucose co-transporter 2. Beta-blockers might prevent tachycardia and/or angina, but they can also produce symptomatic hypotension or fatigue. As we have stated previously, inotropics may provide symptomatic benefit and can facilitate dying at home and avoid rehospitalizations but should be withdrawn in patients approaching the last days/hours and in those without symptomatic benefit. Finally, hypotonia is common in patients approaching the end of life, and might be aggravated by drugs that decease blood pressure. Drug reduction or even withdrawal should be evaluated, particularly in the case of therapies that do not influence symptoms or trajectory of HF, as calcium channel blockers or nitrates.

**Table 2:** Most common adjustment described in the literature of cardiovascular drugs in patients with advanced heart failure in need of palliative care.

	**Recommendation**	**When to reduce dose or withdraw**
Diuretics	Keep unless clear reason to stop	Hypovolemia, hyponatriemia, dehydration, hypotonia
Beta-blockers	Consider gradual dose reduction, risk of reflex tachyarrhythmias	Fatigue, hypotension, bradycardia
ACE inhibitor, ARB, Sacubitril/valsartan, MRA	Keep, consider dose reduction	Hypotension, renal failure, hyperkaliemia
SGLT2 inhibitors	Keep	Renal failure
Ivabradinine	Keep	Bradycardia
Inotropics	Keep if symptomatic benefit and if facilitates dying at home.	Withdraw in the last hours and in those without symptomatic benefit
Statins	Withdraw	Almost always
Antiplatelets	Withdraw unless recent PCI	Almost always
Anticoagulation	Withdraw unless high risk of stroke	Bleeding

*(1) All decisions should be done on individual bases; (2) drugs with long-term effects as statins, aspirin, antihypertensives (and sometimes betablockers) usually should be stopped; (3) Cessation of medications will need sensitive explanation as patients may have been informed that these are lifelong therapies;(4) if severe deterioration and when swallowing becomes difficult: keep only drugs that maintain comfort, as subcutaneous options*.

*ACE, angiotensin-converting enzyme; ARB, angiotensin receptor blocker; MRA, mineralocorticoid receptor antagonist; SGLT2, sodium–glucose co-transporter 2; PCI: percutaneous coronary intervention*.

## Factors to Consider When Withdrawing Cardiovascular Therapies

There are several factors to consider before withdrawing cardiovascular therapies. We have created the mnemonic acronym ADMIRATION that facilitates a correct therapy adjustment (Table 3). It is mandatory to assess the benefit-burden rate as knowing the expected time to benefit is essential in the decision making process as most patients with advanced HF will not leave enough time as to see profit from some drugs (22). A truthful empathic dialogue with the patient to establish specific goals helps the patient and the family to understand the need to modify the current treatment. Drug pharmacokinetics and pharmacodynamics frequently change dramatically in patients with advanced HF, for instance due to gut edema, liver dysfunction, and renal insufficiency. We should always consider specific patient needs, and baseline treatment before drug adjustment, with special attention to polypharmacy as the number of drugs is associated with an increased likelihood of adverse reactions. Resource allocation is also a reason to stop treatments that are not harmful but that might already be non-essential. Finally, in patients with implantable cardioverter defibrillators, the possibility of turning off defibrillation function should be covered (23). The goal is avoidance of suffering caused by high voltage therapies, so antitachycardia and antibradycardia pacing, and resynchronization therapy, that do not produce pain and improve symptoms should be kept.

**Table 3:** Authors recommendation regarding factors to consider when withdrawing cardiovascular therapies.

**♦ Assess benefit-burden rate:** Knowing the time to benefit helps treatment decisions
**♦ Dialogue:** Goals might change. Making short-term goals usually helps
**♦ Metabolism:** Pharmacokinetics, bioavailability, and pharmacodynamics frequently change significantly in advanced HF
**♦ Individualize:** Consider specific needs, problems, and previous treatments
**♦ Renal function:** Little evidence to support HF drugs in patients with severe chronic kidney disease
**♦ Adverse effects:** the number of drugs is associated with an increased likelihood of adverse reactions
**♦ Total cost:** Resource allocation is also a legitimate reason to withdraw non-essential treatments
**♦ Implantable cardioverter defibrillator (ICD):** Consider turning off defibrillation function in ICD carriers
**♦ Other:** Emotional, psychological, and cultural effects of withdrawing drugs
**♦ Natural history/prognosis:** Use of specific heart failure prognostic indexes is recommended

*Please note the mnemonic acronym ADMIRATION*.

*HF, heart failure*.

## Drugs as Cause of Fatigue and Other Symptoms

The phenomenon of incapacitating chronic fatigue is very common in patients with advanced HF. Although this symptom is mainly related with low cardiac output and reduced blood flow through the muscles and brain, multi-organ failure might also play role, including chronic kidney disease, liver failure, and adrenal insufficiency. Regarding drugs, diuretics and beta-blockers or their side effects, such as hyponatremia (24) or circulatory centralization, may also exacerbate fatigue. Especially in very frail people or those approaching end of live, thirst stimulus can be strongly reduced, with an increasing risk of dehydration. Depression is common in this group of patients and may be slightly aggravated by beta-blockers, although recent data do not support a clear association between beta-blocker therapy and depression (25). Beta-blockers may also exacerbate the freezing phenomenon of the limbs as a result of vasoconstrictive influence on microcirculation, a property mainly exhibited by beta-blockers devoid of vasodilatory properties.

Another factor contributing to significant weakness and aggravating the feeling of fatigue is anemia, which may be a consequence of bleeding resulting from the use of antiplatelet or anticoagulant drugs. Bleeding frequently causes worsening HF and rehospitalization (26). Moreover, these drugs are frequently prescribed with proton pump inhibitors. Proton pump inhibitors may increase the risk of cardiovascular events and might increase the risk of Clostridium difficile infection (27, 28).

## Withdrawing Life-Sustaining Drugs

The concept of life-sustaining drugs is not straightforward, although in some patients with advanced HF the decision to stop inotropes might be associated with death in a short period of time. Ethical commitments can make it hard for doctors to consider withdrawing life-sustaining drugs even when the patient, or their family, do not want treatment to be continued (29). Unfortunately, physicians and families often just do not know advanced HF patients' wishes and expectations. A written attestation from the patient and a formal record of drug might improve decision-making regarding drug withdrawal. Withdrawing life-sustaining drugs has clinical aspects but also ethical, legal, cultural, religious, and financial aspects that might also influence this decisions. Before taking the decision, it is essential to understand whether the treatment is providing a benefit to the patient. The term medical futility, although widely used, has recently been deemed misleading and calls have been made for it to be reserved for drugs that have no possibility of working (30). “Potentially inappropriate” are now being used for drugs that have at least some chance of still benefiting the patient, but clinicians believe that competing scientific and ethical considerations justify not to provide them.

## Future Trends

HF prevalence and health loss burden are constantly increasing, especially in the elderly. We need to redesign healthcare access, infrastructure and therapies including a multidisciplinary approach that includes palliative care of patients with advanced HF (31). New tools like telemedicine and artificial intelligence might help in the early detection of symptomatic needs but always with a personalized HF patient-centered approach (32). Despite greater adoption of a palliative approach in the terminal admission over the last decade, a significant proportion of patients with HF keep their usual medical treatment and receive palliative care late, just prior to death (31). An earlier recognition of HF terminal phase, to facilitate a correct modification of cardiovascular drugs and provision of an appropriate palliative approach remains a challenge. The involvement of patients and their care providers is essential to this adjustment of medical treatment. Patients with advanced HF have a high mortality and large healthcare utilization. Programs of integrated care for have been shown to be effective (33) and to increase treatment adjustments and the proportion of HF patients who died in non-acute care settings. More proactive, individualized palliative care referral of advanced HF patients to specialized palliative care should be implemented, particularly in the case of difficult and challenging scenarios.

In this manuscript, we have tried to present the challenges associated with cardiovascular drugs and life sustaining withdrawing. The readers should be aware that specific aspects of clinical practice and law may vary in different countries. Also, although we have focused in deprescribing, the decision not to start a drug is quite similar.

## Conclusions

The adjustment of cardiovascular drugs is frequently needed in patients with advanced HF. The shift in emphasis from life-prolonging to symptomatic treatments should be a gradual one. This is frequently a difficult process for patients and professionals, as drug discontinuing needs to balance treatment benefit with the psychological adaption to having a terminal illness. The golden rule is to stop drugs that are harmful or non-essential and to continue the ones that provide symptomatic improvement. In complex cases, specialized palliative care consultation in the deprescribing process might be needed.

## Author Contributions

MM-S prepared the first draft of the manuscript. TG improved the manuscript with relevant content. Both authors contributed to the article and approved the submitted version.

## Conflict of Interest

The authors declare that the research was conducted in the absence of any commercial or financial relationships that could be construed as a potential conflict of interest.

## Publisher's Note

All claims expressed in this article are solely those of the authors and do not necessarily represent those of their affiliated organizations, or those of the publisher, the editors and the reviewers. Any product that may be evaluated in this article, or claim that may be made by its manufacturer, is not guaranteed or endorsed by the publisher.

## Footnotes

^1^ https://www.who.int/nmh/Global_Atlas_of_Palliative_Care.pdf
